# Propolis, a Constituent of Honey, Inhibits the Development of Sugar Cataracts and High-Glucose-Induced Reactive Oxygen Species in Rat Lenses

**DOI:** 10.1155/2016/1917093

**Published:** 2016-05-08

**Authors:** Teppei Shibata, Shinsuke Shibata, Naoko Shibata, Etsuko Kiyokawa, Hiroshi Sasaki, Dhirendra P. Singh, Eri Kubo

**Affiliations:** ^1^Department of Ophthalmology, Kanazawa Medical University, Ishikawa 920-0293, Japan; ^2^Department of Oncogenic Pathology, Kanazawa Medical University, Ishikawa 920-0293, Japan; ^3^Department of Ophthalmology and Visual Sciences, University of Nebraska Medical Center, Omaha, NE 68198-5540, USA

## Abstract

*Purpose.* This study investigated the effects of oral propolis on the progression of galactose-induced sugar cataracts in rats and the* in vitro* effects of propolis on high-glucose-induced reactive oxygen species (ROS) and cell death in cultured rat lens cells (RLECs).* Methods*. Galactose-fed rats and RLECs cultured in high glucose (55 mM) medium were treated with propolis or vehicle control. Relative lens opacity was assessed by densitometry and changes in lens morphology by histochemical analysis. Intracellular ROS levels and cell viability were measured.* Results*. Oral administration of propolis significantly inhibited the onset and progression of cataract in 15% and 25% of galactose-fed rats, respectively. RLECs cultured with high glucose showed a significant increase in ROS expression with reduced cell viability. Treatment of these RLECs with 5 and 50 *μ*g/mL propolis cultured significantly reduced ROS levels and increased cell viability, indicating that the antioxidant activity of propolis protected cells against ROS-induced damage.* Conclusion*. Propolis significantly inhibited the onset and progression of sugar cataract in rats and mitigated high-glucose-induced ROS production and cell death. These effects may be associated with the ability of propolis to inhibit hyperglycemia-evoked oxidative or osmotic stress-induced cellular insults.

## 1. Introduction

Hyperglycemia is the major factor in the development of diabetic cataracts. Hyperglycemia activates the aldose reductase (AR)/sorbitol (polyol) pathway and induces osmotic and/or oxidative stress [1–6]. AR converts aldose sugars, such as glucose and galactose, to polyols in the presence of reduced nicotinamide-adenine dinucleotide phosphate (NADPH), which is abundant in lens fibers [3, 7]. In the lens, osmotic stress induced by sorbitol accumulation is regarded as the major factor in the progression of diabetic cataract [1–3, 8, 9]. Activation of the polyol pathway also increases oxidative stress by increasing levels of hydrogen peroxide [10] and free radicals [11].

Hyperglycemia-induced oxidative stress has been shown to be a major culprit in the development and progression of galactose-associated cataracts, a process inhibited by antioxidants and AR inhibitors (ARI) [11]. Increased osmotic stress associated with activation of the polyol pathway can induce endoplasmic reticulum stress, generating reactive oxygen species (ROS), which in turn induce cell damage [12]. Hyperglycemia associated ROS are generated by the autoxidation of glucose, the nonenzymatic glycation of proteins, the glucose-induced activation of protein kinase C, increased polyol pathway activity, and impairment of antioxidant enzymes and alterations in mitochondria [13–17]. Taken together, these findings suggested that oxidative stress induced by osmotic stress and hyperglycemia is a major factor in the etiology of cell death signaling that finally leads to cataractogenesis.

Recently, considerable effort has sought to identify therapeutic agents, including those of phytochemical origin, which can promote naturally occurring cellular antioxidant defense systems. Propolis is a component of honey collected by bees from tree buds and widely available as a dietary supplement. Propolis and caffeic acid phenethyl ester (CAPE), an active component of propolis extract, have been shown to have immunomodulatory, antitumor, cytotoxic, antimetastatic, anti-inflammatory, and antioxidant properties [18]. Moreover, propolis has been shown to normalize glucose homeostasis in animal models of type 2 diabetes [19, 20].

However, propolis contents and activity have been associated with specific plant sources, collecting location and phytogeographic areas. Several studies have investigated the activity against various disorders, including hyperglycemia, of propolis obtained from different geographical regions. To date, however, the ability of propolis to delay or inhibit sugar or oxidative stress-induced cataract formation has not been investigated. This study therefore examined the* in vivo* effect of orally administered green Brazilian propolis on the development of galactose-induced cataract in rats and the* in vitro* effects of propolis on high-glucose-induced ROS levels and cell viability in cultured rat lens epithelial cells (RLECs). The results of this study may contribute to the development of strategies to prevent or delay hyperglycemia-induced complications.

## 2. Materials and Methods

### 2.1. Cell Culture

RLECs isolated from SD rats were cultured in Dulbecco's modified eagle medium (DMEM; Sigma, St. Louis, MO, USA), supplemented with 20% fetal bovine serum (FBS; Sigma) at 37°C in an air/CO_2_ (19 : 1) atmosphere. At passages 8–10, the medium was replaced by serum-free medium containing 50 *μ*g/mL purified honey or 5 or 50 *μ*g/mL propolis and cultured for 12 hrs. The cells were subsequently cultured for 120 hrs in high glucose medium, consisting of DMEM, 5% FBS, and 5.5 mM or 55 mM D-glucose (Sigma) or, as osmotic control, 55 mM D-mannitol (Sigma) and with 50 *μ*g/mL purified honey or 5 or 50 *μ*g/mL propolis.

### 2.2. Quantitation of Intracellular ROS

Intracellular ROS level was measured using the fluorescent dye, dichlorofluorescein diacetate (H2-DCF-DA), and a nonpolar compound converted to a polar derivative (dichlorofluorescein) by cellular esterase after incorporation into cells [21]. RLECs (5 × 10^3^) were cultured in 96-well plates for 120 hrs with DMEM and 5% FBS; 5.5 mM, 55 mM D-glucose, or 55 mM D-mannitol; and 50 *μ*g/mL purified honey or 5 or 50 *μ*g/mL propolis.

The medium was replaced with Hank's solution (Sigma) containing 10 *μ*M DCFH-DA (Cayman Chemical, Ann Arbor, MI) and incubated for 10 min at room temperature. Intracellular fluorescence was detected at an excitation wavelength of 485 nm and an emission wavelength of 530 nm using Spectra Max Gemini EM (Molecular Devices, Sunnyvale, CA).

### 2.3. Cell Viability Assay (MTS Assay)

Colorimetric MTS assays (Promega, Madison, WI, USA) were performed as described [21]. Briefly, RLECs (5 × 10^3^) were cultured in 96-well plates for 120 hrs in DMEM and 5% FBS; 5.5 mM and 55 mM D-glucose or 55 mM D-mannitol; and 50 *μ*g/mL purified honey or 5 or 50 *μ*g/mL propolis. To each well was added 3-(4,5-dimethylthiazol-2-yl)-5-(3-carboxymethoxyphenyl)-2-(4-sulfophenyl)-2H-tetrazolium salt (MTS) for 4 hrs. In this assay, MTS is reduced to a water soluble formazan salt when added to medium containing viable cells, with this reduction determined by measuring absorbance at 490 nm with a microplate reader. Results were normalized relative to the absorbance of untreated control(s).

### 2.4. Animals

All animal experiments were approved by the Committee of Animal Research at Kanazawa Medical University (Permission number: 2013-88) and were conducted in accordance with the National Institutes of Health Guide for the Care and Use of Laboratory Animals, the recommendations of the Association for Research in Vision and Ophthalmology (ARVO) Statement on the Use of Animals in Ophthalmic and Vision Research, and the Institutional Guidelines for Laboratory Animals of Kanazawa Medical University.

Seven-week-old female Sprague-Dawley (SD) albino female rats were obtained from Sankyo Labo Service Corporation Inc. (Toyama, Japan). Purified honey and 30% water soluble Brazilian propolis were supplied by Yamada Bee Farm Corporation (Okayama, Japan). Prior to treatment, all rats were given ad libitum access to regular chow (Oriental Yeast, Osaka, Japan). Rats were subsequently fed 0.6 g/kg purified honey (*n* = 15), 0.1 g/kg propolis (*n* = 10), or 0.6 g/kg propolis (*n* = 10) every day for 1 week. To induce sugar cataracts, these rats were given ad libitum access to 15% or 25% D-galactose mixed with regular chow (Oriental Yeast), as well as being continued on 0.6 g/kg/day purified honey, 0.1 g/kg/day propolis, or 0.6 g/kg/day propolis, for 3 weeks. Control rats were allowed ad libitum access to regular chow, as well as being continued on 0.6 g/kg/day purified honey, for 3 weeks. The rats were subsequently sacrificed and lenses from the right eye of each were carefully removed and photographed using a stereomicroscope under dark-field illumination (Zeiss, Stemi DV4, Jena, Germany). The density of opacity was analyzed using MultiGauge Software (Fuji Film, Tokyo, Japan). Left eyes were processed for paraffin sectioning, stained with hematoxylin and eosin (H&E) and examined histologically.

### 2.5. Statistical Analysis

The results are reported as means ± standard deviation and were analyzed statistically using ANOVA with Fisher's test.

## 3. Results

### 3.1. Effect of Propolis on High-Glucose-Induced ROS Production and Cell Survival

**Figure 1:**
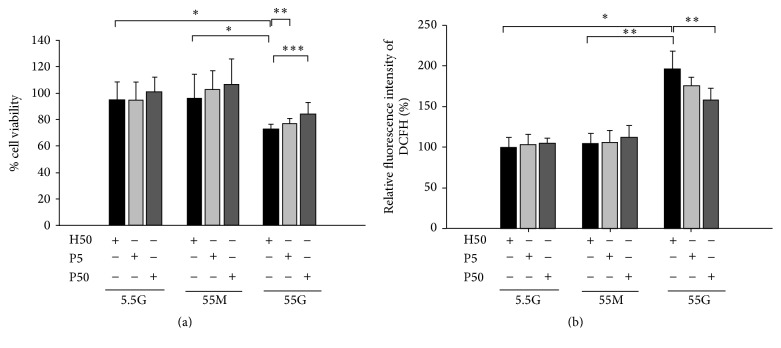
Effect of propolis on the viability and ROS production of cultured RLECs. Effect of propolis on intracellular ROS production and survival of RLECs following high glucose stress. Honey (50 *μ*g/mL; H50) or propolis (5 or 50 *μ*g/mL; P5 or P50) was added to RLECs cultured with 5.5 or 55 mM D-glucose or with 55 mM D-mannitol as osmotic control; 120 hrs later, ROS levels were measured using H2-DCFH-DA assay (a) and cell viability was estimated using colorimetric MTS assay (b). Results shown are the mean ± SD of three experiments. Asterisks denote statistically significant differences. (a) ^*∗*^ *P* < 0.009, ^*∗∗*^ *P* < 0.05, and ^*∗∗∗*^ *P* < 0.002. (b) ^*∗*^ *P* < 0.0000001 and ^*∗∗*^ *P* < 0.04.

To determine if propolis could inhibit high-glucose-induced ROS and reduced survival of RLECs cultured* in vitro*, RLECs cultured in DMEM containing 5.5 mM and 55 mM D-glucose or 55 mM D-mannitol (Figure 1) were treated with 5 or 50 *μ*g/mL propolis or 5 or 50 *μ*g/mL purified honey. Cell viability assays showed that culture with 50 mM glucose significantly reduced cell survival but that the addition to these cells of 5 or 50 *μ*g/mL propolis significantly enhanced their viability, more so than that of RLECs cultured with 50 mM glucose and treated with 50 *μ*g/mL honey (Figure 1(a)). Quantitation of ROS levels using H2-DCF-DA showed increased ROS levels in RLECs treated with 50 mM glucose (Figure 1(b)), whereas the addition to these cells of 5 or 50 *μ*g/mL propolis resulted in significantly lower ROS levels than the addition of 50 *μ*g/mL honey (Figure 1(b)).

### 3.2. Animals and Their Characteristics

Table 1 shows the body weight of normal (control) rats and rats fed 15% or 25% galactose in the presence or absence of 0.1 or 0.6 g/kg/day water soluble propolis or 0.6 g/kg/day honey. Although body weights did not differ significantly at the beginning of propolis or honey administration (*P* > 0.05), they increased in all three groups after 3 weeks (Table 1). Body weights of rats fed 25% galactose plus 0.1 or 0.6 g/kg/day propolis for 3 weeks were significantly lower than the body weights of control rats fed 0.6 g/kg/day honey (*P* < 0.02). This reduction was due to galactose, as body weights were similar in rats fed 25% galactose plus 0.1 g/kg/day honey or 0.1 or 0.6 g/kg/day propolis.

### 3.3. Propolis Inhibition of Sugar Cataracts

**Figure 2:**
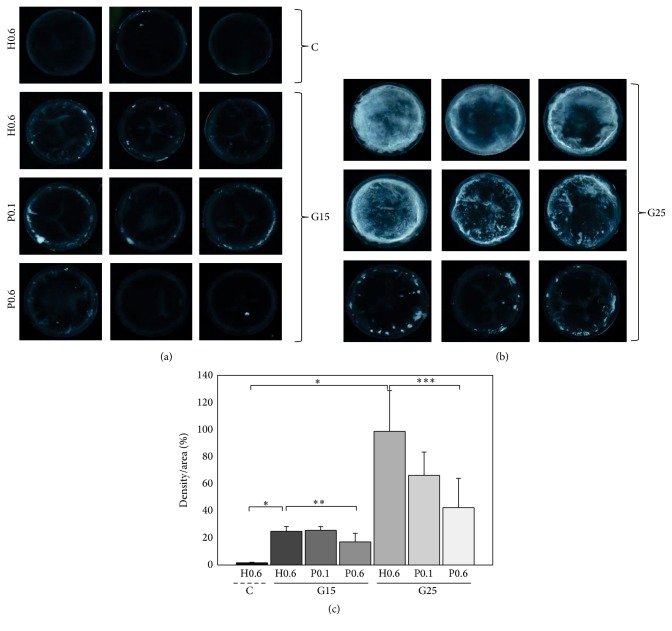
Effect of water soluble propolis on lens opacity in rats fed a high galactose diet. Rats in each group were given ad libitum access to 15% or 25% D-galactose mixed with regular chow, as well as being continued on 0.6 g/kg/day purified honey, 0.1 g/kg/day propolis, or 0.6 g/kg/day propolis, for 3 weeks. Control rats were allowed ad libitum access to regular chow, as well as being continued on 0.6 g/kg/day purified honey, for 3 weeks. Propolis suppressed lens opacity in rats fed (a) 15% and (b) 25% galactose. (c) Densitometry shows that oral intake of propolis (0.6 g/kg) significantly suppressed lens opacity in galactose-fed rats. Results shown are the mean ± SD of three experiments. Asterisks denote statistically significant differences. ^*∗*^ *P* < 0.000006; ^*∗∗*^ *P* < 0.05; ^*∗∗∗*^ *P* < 0.01.

To determine if propolis was effective in delaying or preventing cataract progression or formation, lens opacification was analyzed in propolis- and honey-treated rats. Cortical and supranuclear opacity were observed in rats fed 15% and 25% galactose, with the severity of lens opacity being greater in rats fed 25% galactose (Figure 2(a)). Administration of 0.6 g/kg/day propolis significantly reduced lens opacity (Figures 2(a) and 2(b)), indicating that water soluble propolis inhibits sugar-induced cataractogenesis in rats.

**Figure 3:**
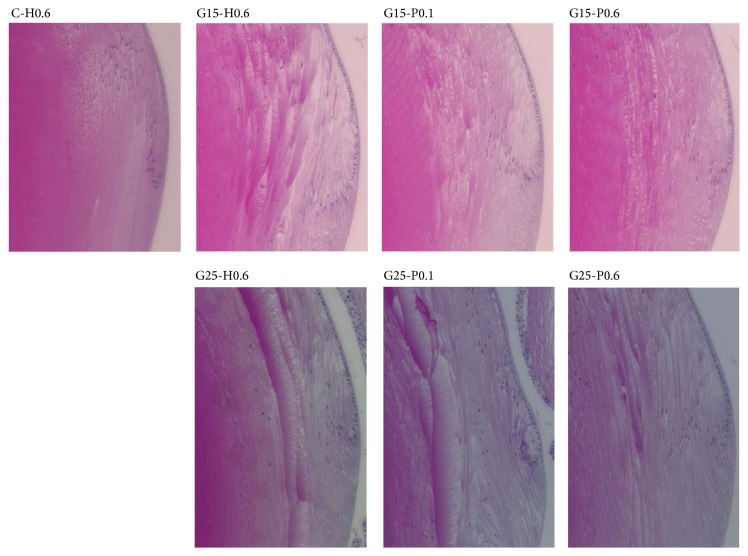
Histological images of sugar cataract formation in rats fed diets containing 15% and 25% galactose with/without propolis treatment. Rats fed a normal diet plus 0.6 g/kg/day honey showed no cataractous changes ((a) and (b)). Three weeks after feeding with 15% galactose plus 0.6 g/kg/day honey, lens fiber swelling was observed in the anterior and equatorial regions, whereas feeding with 25% galactose plus 0.6 g/kg/day honey resulted in marked swelling and liquefaction of the cortical fibers in the equatorial region of the lenses. These histological changes were suppressed by treatment with 0.6 g/kg/day propolis.

Histopathological analysis showed no detectable histological changes in the lenses of rats fed a control diet with 0.6 g/kg/day honey (Figure 3). However, some peripheral opacity was observed in the equatorial and cortical regions of rats fed 15% galactose plus 0.6 g/kg/day honey. Swelling of lens fibers was reduced in rats fed 15% galactose plus 0.1 g/kg/day propolis and was significantly inhibited in rats fed 15% galactose plus 0.6 g/kg/day propolis, with the latter also showing small vacuole formation between lens fibers. Rats fed 25% galactose plus 0.6 g/kg/day honey showed progression of lens swelling, with deeper lesions and liquefaction of the cortex. Administration of 0.6 g/kg/day propolis reduced lens fiber swelling in rats fed 25% galactose, whereas administration of 0.1 g/kg/day propolis did not.

## 4. Discussion

This study evaluated the ability of water soluble propolis to inhibit or delay the development of sugar- and oxidative stress-induced cataractogenesis. We found that propolis could inhibit high-glucose-induced ROS generation and cell death in cultured RLECs. We also found that propolis inhibited the formation of galactose-induced cataracts. The polyol pathway has been reported to be responsible for generating osmotic stress and oxidative stress [10, 11, 22, 23], with antioxidant treatment postponing the progression of cataracts in diabetic rats [24], thus demonstrating that oxidative stress is a major factor in the chronic development of diabetic cataracts. We have reported that hyperglycemic apoptosis of LECs involves polyol pathway-dependent osmotic stress and oxidative stress [11, 25]. AR upregulation in diabetic cataracts induces apoptosis, demonstrating the therapeutic potential of Prdx6 as an antioxidant in the prevention and control of hyperglycemia-induced complications [25].

Propolis, a resinous substance collected by honeybees from buds and crevices in the bark of various trees, contains more than 200 different constituents, including active organic compounds, such as artepillin C (APC), benzoic acids, flavonoids, caffeoylquinic acids (CAs), cinnamic acid, and derivatives of coumaric acid [26, 27]. CAs were shown to inhibit rat AR activity* in vitro* and the formation of advanced glycation end products and associated protein cross-linking; moreover, administration of CAs prevented the development of sugar cataracts by inhibiting AR activity [28, 29]. Natural flavonoids, such as quercetin, have antioxidant activity and inhibit AR and advanced glycation, suggesting that they may prevent the formation of diabetic cataracts [30, 31]. Although the pure form of each component of propolis should be examined for protection against sugar cataracts and high-glucose-induced ROS production, the ability of propolis to inhibit sugar cataracts in this study may have been due to the ability of CAs and flavonoids to inhibit AR activity. Efforts are needed to identify the constituents of propolis most effective in protecting against ROS and sugar cataracts.

Treatment with 0.1 or 0.6 g/kg/day propolis significantly reduced the body weights of rats fed 25% galactose, compared with control rats treated with 0.6 g/kg/day honey; however, there was no difference in body weight between control rats and 25% galactose-fed rats treated with honey 0.6 g/kg/day/honey. Treatment of obese C57BL/6N mice with propolis extract was found to reduce body weight, the serum concentrations of nonesterified fatty acids, and lipid accumulation in the liver [32]. However, as 25% galactose itself may have reduced body weight, we could not conclude that propolis had an effect on body weight in galactose-fed rats.

In conclusion, this study showed that soluble green propolis can inhibit galactose-induced cataracts in rats. Propolis may reduce oxidative stress and may inhibit the polyol pathway, thus delaying and/or blunting the formation of sugar-induced cataracts.

## Figures and Tables

**Table 1:** Effect of water soluble propolis treatment on body weight in rats with galactose-feeding.

Groups	0 W (g)	3 W (g)
C + H0.6	107.800 ± 11.432	200.400 ± 4.159
G15 + H0.6	102.800 ± 10.208	196.200 ± 10.803
G15 + P0.1	109.600 ± 6.878	199.200 ± 10.450
G15 + P0.6	114.600 ± 9.290	192.200 ± 10.085
G25 + H0.6	106.800 ± 6.340	192.600 ± 8.264
G25 + P0.1^*∗*^	104.400 ± 5.727	188.800 ± 5.762
G25 + P0.6^*∗*^	110.400 ± 9.762	189.400 ± 6.804

All results are expressed as the means ± standard deviations.

C, control diet; G15, diet containing 15% galactose; G25, diet containing 25% galactose; H0.6, administration of 0.6 g/kg/day honey; P0.1, administration of 0.1 g/kg/day propolis; P0.6, administration of 0.6 g/kg/day propolis. ^*∗*^ *P* < 0.02.
